# Brominated flame retardants in placental tissues: associations with infant sex and thyroid hormone endpoints

**DOI:** 10.1186/s12940-016-0199-8

**Published:** 2016-11-25

**Authors:** Christopher Leonetti, Craig M. Butt, Kate Hoffman, Stephanie C. Hammel, Marie Lynn Miranda, Heather M. Stapleton

**Affiliations:** 1 Nicholas School of the Environment, Duke University, 9 Circuit Drive, Box 90328, Durham, NC 27708 USA; 2 Department of Statistics, Rice University, Houston, TX USA

**Keywords:** Polybrominated diphenyl ether (PBDE), 2,4,6-tribromophenol (2,4,6-TBP), Placenta, Thyroid hormone

## Abstract

**Background:**

Brominated flame retardants (BFRs) are endocrine disruptors that bioaccumulate in the placenta, but it remains unclear if they disrupt tissue thyroid hormone (TH) metabolism. Our primary goal was to investigate associations between placental BFRs, TH levels, Type 3 deiodinase (DIO3) activity and TH sulfotransferase (SULT) activities.

**Methods:**

Placenta samples collected from 95 women who delivered term (>37 weeks) infants in Durham, NC, USA (enrolled 2010–2011) were analyzed for polybrominated diphenyl ethers (PBDEs), 2,4,6-tribromophenol (2,4,6-TBP), THs (T4, T3 and rT3), and DIO3 and TH SULT activities.

**Results:**

PBDEs and 2,4,6-TBP were detected in all placenta samples. PBDEs were higher in placental tissues from male infants compared to female infants, with 2,4,6-TBP and BDE-209 levels approximately twice as high. Among male infants, placental BDE-99 and BDE-209 were negatively associated with rT3 placental levels. For female infants, placental BDE-99 and 2,4,6-TBP were positively associated with T3 concentrations. DIO3 activity was also significantly higher in placental tissues from male infants compared to females, while 3,3’-T2 SULT activity was significantly higher in placental tissues from females compared to males. Among males, several PBDE congeners were positively correlated with T3 SULT, while BDE-99 was negatively associated with T3 SULT among females. Associations generally remained after adjustment for potential confounding by maternal age and gestational age at delivery.

**Conclusions:**

These results suggest BFRs accumulate in the placenta and potentially alter TH function in a sex-specific manner, a possible mechanism to explain the sex-dependent impacts of environmental exposure on children’s growth and development. More research is needed to elucidate the effects of BFRs on placenta function during pregnancy, as well as the biological consequences of exposure and thyroid disruption.

**Electronic supplementary material:**

The online version of this article (doi:10.1186/s12940-016-0199-8) contains supplementary material, which is available to authorized users.

## Background

Brominated flame retardants (BFRs) such as polybrominated diphenyl ethers (PBDEs) and 2,4,6-tribromophenol (2,4,6-TBP) have been applied to numerous types of furniture and electronic items in order to meet state and federal flammability standards [1]. PBDE mixtures were phased out of use and production because of their persistence, bioaccumulation, and potential toxicity beginning in 2004 [2, 3]. Despite their phase out, many older products containing PBDEs remain in use in the home environment, suggesting human exposure to PBDEs will continue for some time. 2,4,6-TBP is an intermediate in the production of other BFRs and also has been used as a reactive flame retardant. In addition, 2,4,6-TBP is currently used as fungicide/wood preservative. Although less is known about 2,4,6-TBP, data indicate that human exposure is ubiquitous and, like PBDE exposure, ongoing [4, 5].

PBDEs and 2,4,6-TBP have similar chemical structures (i.e. the presence of a hydroxyl group and halogens on an aromatic ring) to thyroid hormones (THs) leading to concerns about their potential impact on TH regulation, an important process with numerous down-stream health impacts. Indeed, several studies demonstrate that PBDEs and 2,4,6-TBP disrupt hormone regulation in birds, rodents, and fish [6–9] and epidemiological studies indicate that PBDE exposure impacts thyroid hormone regulation [10]. Proposed mechanisms of TH dysfunction include disruption of TH serum transporters such as transthyretin (TTR) and thyroid-binding globulin (TBG) via the displacement of endogenous THs, aberrant binding to TH nuclear receptors, or disruption of TH-metabolizing enzymes such as deiodinases (DIO) and sulfotransferases (SULT) [11–18], but these effects may be tissue specific, and remain to be fully elucidated.

THs are known to be particularly important for fetal growth and development [19]. During pregnancy, the placenta facilitates the uptake of THs from maternal circulation and subsequent delivery to the fetal compartment, particularly during the first trimester, when the fetus relies solely on maternally-derived THs [20]. TH delivery to target cells involves numerous active and passive transport pathways, but is regulated in part by the activity of TH metabolizing enzymes in the placenta. In our previous in vitro work, we demonstrated that the activities of TH-metabolizing enzymes (e.g. DIO and SULT) are inhibited by exposure to PBDEs, 2,4,6-TBP and other halogenated compounds [11, 12], suggesting that they may impact placental TH concentrations and fetal TH delivery. Deiodinase type 3 (DIO3) in particular is highly expressed in the placenta, and its primary function is to convert thyroxine (T4) to the genomically inactive hormone, reverse triiodothyronine (rT3). This action serves to both buffer the levels of T4 reaching the fetus, and also provide a source of iodine to the fetus [21, 22]. While BFRs have been detected in human placenta samples previously (e.g. [23–25]), the clinical significance of these measures, and their potential importance in thyroid hormone regulation have not, to our knowledge, been evaluated.

The primary goal of this study was to examine associations between BFRs (PBDEs and 2,4,6-TBP), TH concentrations, and measures of TH metabolic enzyme activity in human placental tissues. Based on our previous work in pooled hepatic microsomes, we hypothesized that BFR exposure may be associated with decreased TH enzyme function (e.g. DIO3) which may result in increased (e.g. T4) or decreased (e.g. rT3) TH levels in placental tissues. Such changes may be important for understanding life-long health risks of exposure to PBDEs and 2,4,6-TBP, particularly as the disruption of TH regulation can lead to effects that may manifest later in life as cognitive and behavioral deficits and altered growth [19].

## Methods

### Study population

Participants were recruited within a prospective cohort study assessing the joint effect of social, environmental, and host factors on pregnancy outcomes (the Healthy Pregnancy, Healthy Baby (HPHB) Study). The HPHB study enrolled pregnant women from the Duke Obstetrics Clinic and the Durham County Health Department Prenatal Clinic [26–28]. Our analyses included a subset of women from the HPHB study who were seen at the latter clinic, delivered at the Duke University Medical Center between March 2010 and December 2011, and from whom sufficient placenta tissue was available for this study (*n* = 102). Because we anticipated that gestational age may be related to TH concentrations in placenta, we further restricted analyses to women giving birth to term infants (37 or more weeks gestation (*n* = 95)). Reflective of the population of women receiving services at the clinic used in recruitment, the HPHB cohort is predominantly African-American with the majority of mothers having a high school diploma or less education and few having private health insurance [23]. All aspects of this study were carried out in accordance with a human subjects research protocol approved by the Duke University Institutional Review Board and all women provided informed consent prior to participation.

### Placenta BFR and thyroid hormone analysis

Placenta tissue subsamples were taken at the time of delivery at the Duke University Medical Center. Tissues (approximately 5–20 g) were stored in screwtop cryovials at −80 °C until analysis. BFR levels in these placental tissues were previously described (see [23]) and methods are therefore not reported here. All sample values were blank subtracted and method detection limits (MDLs) were calculated as three times the standard deviation of the lab blank values for each analyte. TH levels were measured using a modified method from our laboratory; complete details on this method can be found in the Additional file 1: Supplemental material [29, 30].

### Preparation of placental microsomes/cytosol and deiodinase/sulfotransferase activity assays

Preparation of placental microsomes and DIO3 assays were performed using a modified method from our laboratory [12, 30]. Preparation of placental cytosolic fractions and SULT assays were performed using a modified method from our laboratory [11]. A detailed set of protocols are provided in the Additional file 1: Supplemental material.

### Quality control/Quality assurance

For QA/QC in the TH measurements, lab blanks were run in each batch of placenta tissue sample extractions, and isotopically labeled internal standards were used in all samples. All sample values were blank subtracted and method detection limits (MDLs) were calculated as three times the standard deviation of the lab blank values for each analyte. All samples were run with 5 ng ^13^C-T3, ^13^C-rT3, and ^13^C-T4 as internal standards. The recovery of the ^13^C-labeled THs were calculated for all tissue samples and lab blanks in order to assess the recovery efficacy of the extraction and clean-up methods (mean blank recovery of THs is 101%; mean sample recovery of THs is 75%).

For 3,3’-T2 SULT assays, all placenta cytosol samples were analyzed in duplicate. For T3S SULT assays, each batch of ten placenta cytosol samples included one duplicate sample to assess assay precision.

### Statistical analysis

Preliminary analyses (Shapiro-Wilkes Test) indicated that TH, BFR, and enzyme activity data were not normally distributed. Accordingly, non-parametric statistical tests were used or data were log_10_ transformed prior to statistical analyses. We used Spearman correlation coefficients to assess associations between BFRs and THs. To assess factors associated with placenta BFRs and relationships between BFRs and THs, we used linear regression models (continuous outcome measures were log_10_-transformed). To aid in the interpretation of regression results, we exponentiated beta coefficients (10^β^), producing the multiplicative change in outcome. As predictors of THs and enzyme activity, BFR levels were categorized into tertiles in order to minimize the effect of skewed data and outliers. Given the lower detection frequency of BDE-209, data were dichotomized into those with detectable levels of BDE-209 and non-detects. Regression analyses were also adjusted a priori for maternal age and gestational age at delivery, factors which we anticipated might confound associations between BFRs and THs. In addition, we considered potential confounding by maternal pre-pregnancy BMI and race; however, the inclusion of these variables did not impact regression coefficients (change of less than 10%) and they were not included in the final adjustment set.

Increasingly the epidemiologic literature suggests that prenatal exposure to environmental chemicals impacts male and female infants differently. We hypothesized that there may be important sex differences in associations between BFRs, THs and enzyme activity. To explore these differences we conducted all analyses in the full cohort (combined), as well as stratified by infant sex. All sex-specific analyses were conducted using *n* = 94 because the sex of the infant was missing for one sample. Statistical analyses were performed using SAS Version 9.2. A *p*-value of 0.05 was considered statistically significant.

### Maternal serum

The primary focus of our work was to evaluate relationships between placental BFRs, TH concentrations, and measures of TH metabolic enzyme activity; however, maternal 3^rd^ trimester serum samples were previously assessed for BFRs for a subsample of mother’s in our current cohort (*n* = 80 matched pairs). Details of these samples have been described previously in [31]. Although these data were not available for our full cohort, we did evaluate relationships between maternal serum BFR levels and placenta serum BFR measures (Spearman correlations). In addition, we evaluated potential differences in maternal serum BFR levels by infant sex. Analyses were limited to BDE-47, BDE-100 and BDE-153 which were all detected in >70% of serum samples. Other congeners were detected less frequently. Within the subsample geometric means of these congeners were 12.42 ng/g lipid for BDE-47, 2.64 ng/g lipid for BDE-100, and 1.48 ng/g lipid for BDE-153.

## Results

### Population characteristics

Of the 95 women included in analyses, 68% were non-Hispanic black, and 57% were under 25 years old. This was the first pregnancy for 45.5% of the women, and 43.6% of the women had completed more than a high school education. Finally, less than 10% of the women had private health insurance. Additional information on the demographics of this population have been reported in [23].

### Brominated flame retardants

**Fig. 1:**
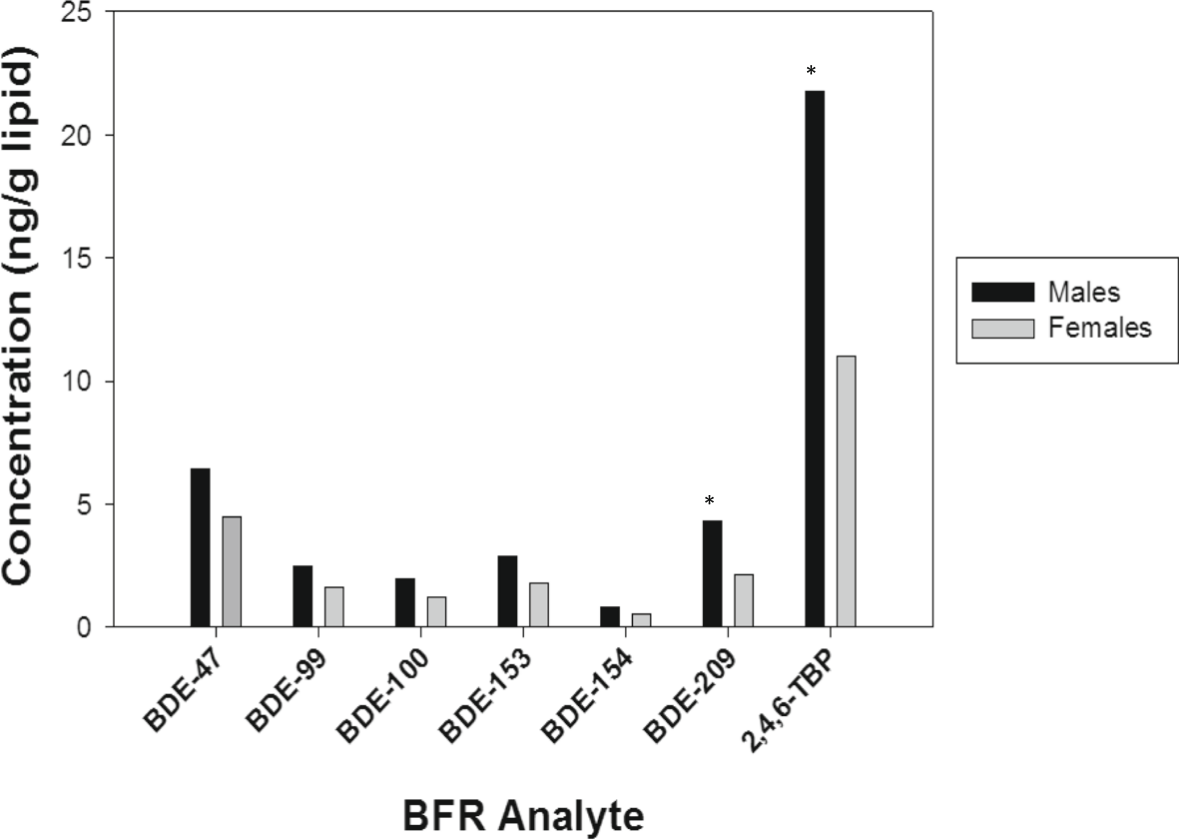
Geometric mean placenta BFR concentrations by infant sex. (* indicates *p* < 0.05; *n* = 94)

Detailed information on the concentrations of the different PBDE congeners and 2,4,6-TBP have been reported previously [23]. Briefly, detection frequencies for BDE-47, −100, −99, −154, −153, −209, and 2,4,6-TBP were all > 50%. The most common PBDE measured was BDE-47, representing 34% of ΣBDE burden. Generally, 2,4,6-TBP was detected at higher concentrations than the PBDEs; the geometric mean concentration of 2,4,6-TBP was 15.4 ng/g lipid (range: 1.31–316 ng/g lipid), while the geometric mean concentration of BDE-47 was 5.09 ng/g lipid (range: 0.12–141 ng/g lipid). Concentrations were higher in placental tissues associated with male infants compared to female infants (Fig. 1). For example, BDE-209 and 2,4,6-TBP were approximately twice as high in placental tissues associate with male infants as compared to female infants (*p* <0.01). In samples for which we had paired maternal serum (*n* = 80), there was no relationship between maternal serum BDE concentrations and infant sex (serum BDE-47, *p* = 0.59; BDE-100, *p* = 0.62; BDE-153, *p* = 0.72); despite their being significant, or near significant, differences by sex in placental BFRs concentrations in the subsample (placenta BDE-47, *p* = 0.06; BDE-100, *p* = 0.05; BDE-153, *p* = 0.04). Interestingly, the only significant correlation between serum and placenta BDE levels was for BDE-47 (*r*_s_ = 0.36, *p* = 0.009); correlations for BDE-100 and BDE-153 were smaller and not statistically significant.

### Thyroid hormones

Thyroid hormones were detected in 100% of the placenta tissue samples. Descriptive statistics for the TH measurements are provided in Table 1. T4 was measured in the highest concentration with a geometric mean of 28.1 ng/g ww (range: 11.8–53.6 ng/g ww), followed by rT3 with a geometric mean of 2.64 ng/g ww (range: 0.73–7.59 ng/g ww), and T3 was measured in the lowest concentrations with a geometric mean of 0.37 ng/g ww (range: 0.10–0.84 ng/g ww). Levels of T4, T3, or rT3 in placental samples did not differ by infant sex (results not shown).

**Table 1:** Distribution or BFRs, THs and enzyme activity in placenta samples (*n* = 95)

					Percentile		
Variable	MDL	Detection Frequency (%)	Geometric Mean	Min	33rd	67th	Max
PBDEs							
BDE-47	0.07	91.2	5.45	0.12	2.81	10.5	140.5
BDE-99	0.07	68.6	2.02	0.16	1.35	3.47	222.8
BDE-100	0.02	88.2	1.56	0.06	0.86	3.02	50.1
BDE-153	0.01	93.1	2.23	0.07	1.69	3.38	513.2
BDE-154	0.01	83.3	0.64	0.01	0.44	1.07	20.2
BDE-209	0.17	52.9	3.08	0.25	1.94	5.19	50.4
ΣPBDEs			14.6	0.62	8.17	24.0	521.8
Phenolic compound							
2,4,6-TBP	0.05	100	15.8	1.31	7.83	22.7	316.1
ΣBFRs			39.1	2.18	24.0	57.2	568.3
Thyroid hormones							
T4	0.002	100	28.1	11.8	25.1	32.6	53.6
T3	0.002	100	0.37	0.10	0.32	0.45	0.84
rT3	0.002	100	2.64	0.73	2.19	3.25	7.59
Enzyme activity							
DIO3	NA	100	0.74	0.01	0.58	1.12	3.86
3,3’-T2 SULT activity	NA	100	3.32	0.35	2.34	4.59	19.2
T3 SULT activity	NA	100	9.50	3.59	7.90	10.9	37.9

Brominated flame retardant concentrations (ng/g lipid), thyroid hormone levels (ng/g ww), DIO3 activity (pmol rT3/mg protein/min), 3,3’-T2 SULT activity (pmol T2S/mg protein/min), and T3 SULT activity (fmol T3S/mg protein/min) measured in placenta tissue (*n* = 95). MDL indicates method detection limit. *N* = 95

In analyses including all placental tissues (i.e. males and females), BFRs were not correlated with placental T3 or T4 concentrations. However, because we anticipated that the impact of BFRs on TH might vary by infant sex, we examined associations stratified by sex. Among males (*n* = 48), patterns of association were negative between T3 and BDE-99, 2,4,6-TBP, and ΣBFRs; although none were statistically significant. Conversely, among females (*n* = 46), a positive association was observed between T3 and BDE-99, 2,4,6-TBP, and ΣBFRs, and, although not statistically significant, BDE-47, −153, and ΣBDEs also showed a positive trend with T3. Data suggest that T4 may be inversely correlated with BDE-99 and BDE-100 among males; however, there was no clear trend between T4 and BFR concentrations among females. While concentrations of rT3 in placental tissue were significantly inversely correlated with BDE-99 for both males and females, a similar inverse association between rT3 and BDE-209 was only observed among males (*r*_s_ = −0.35; *p* = 0.01; Table 2).

**Table 2:** Spearman correlations between BFRs, thyroid hormones, and enzyme activity by infant sex

		BDE-47	BDE-99	BDE-100	BDE-153	BDE-154	BDE-209	2,4,6-TBP	ΣBFR	ΣBDE	T3	rT3	T4	DIO3	T2S
Males (*n* = 48)	T3	−0.07	−0.21	−0.16	−0.03	−0.13	−0.10	−0.18	−0.22	−0.16	1.00				
	rT3	0.02	−0.34*	0.01	0.13	−0.04	−0.35*	−0.02	−0.13	−0.11	0.14	1.00			
	T4	−0.14	−0.21	−0.21	0.04	−0.07	−0.15	−0.11	−0.16	−0.15	0.53*	0.35*	1.00		
	DI	0.03	0.16	0.15	0.04	−0.05	−0.02	0.00	0.05	0.09	−0.05	0.05	−0.27	1.00	
	T2S	0.02	−0.14	0.07	−0.01	0.02	−0.14	−0.02	−0.05	−0.01	−0.13	0.29*	−0.15	0.11	1.00
	T3S	0.29*	0.03	0.26^#^	0.27^#^	0.16	0.00	−0.11	−0.02	0.25^#^	−0.15	0.10	−0.05	0.17	0.29
Females (*n* = 46)	T3	0.22	0.33*	0.19	0.23	0.08	0.17	0.36*	0.35*	0.25^#^	1.00				
	rT3	−0.12	−0.34*	−0.08	−0.10	−0.07	−0.05	−0.14	−0.13	−0.19	−0.16	1.00			
	T4	0.08	−0.07	−0.02	−0.03	−0.06	0.06	0.06	0.10	0.03	0.45*	0.24	1.00		
	DI	0.10	0.00	0.18	0.11	0.24^#^	0.22	0.02	0.08	0.09	−0.10	0.23	0.02	1.00	
	T2S	0.11	−0.06	0.05	0.13	0.11	−0.10	−0.11	0.02	0.13	−0.19	−0.08	−0.12	−0.16	1.00
	T3S	0.08	−0.25^#^	0.11	0.15	0.15	−0.02	0.02	0.05	0.05	−0.07	−0.06	0.05	0.17	0.19

* < 0.05, ^#^ < 0.10

**Fig. 2:**
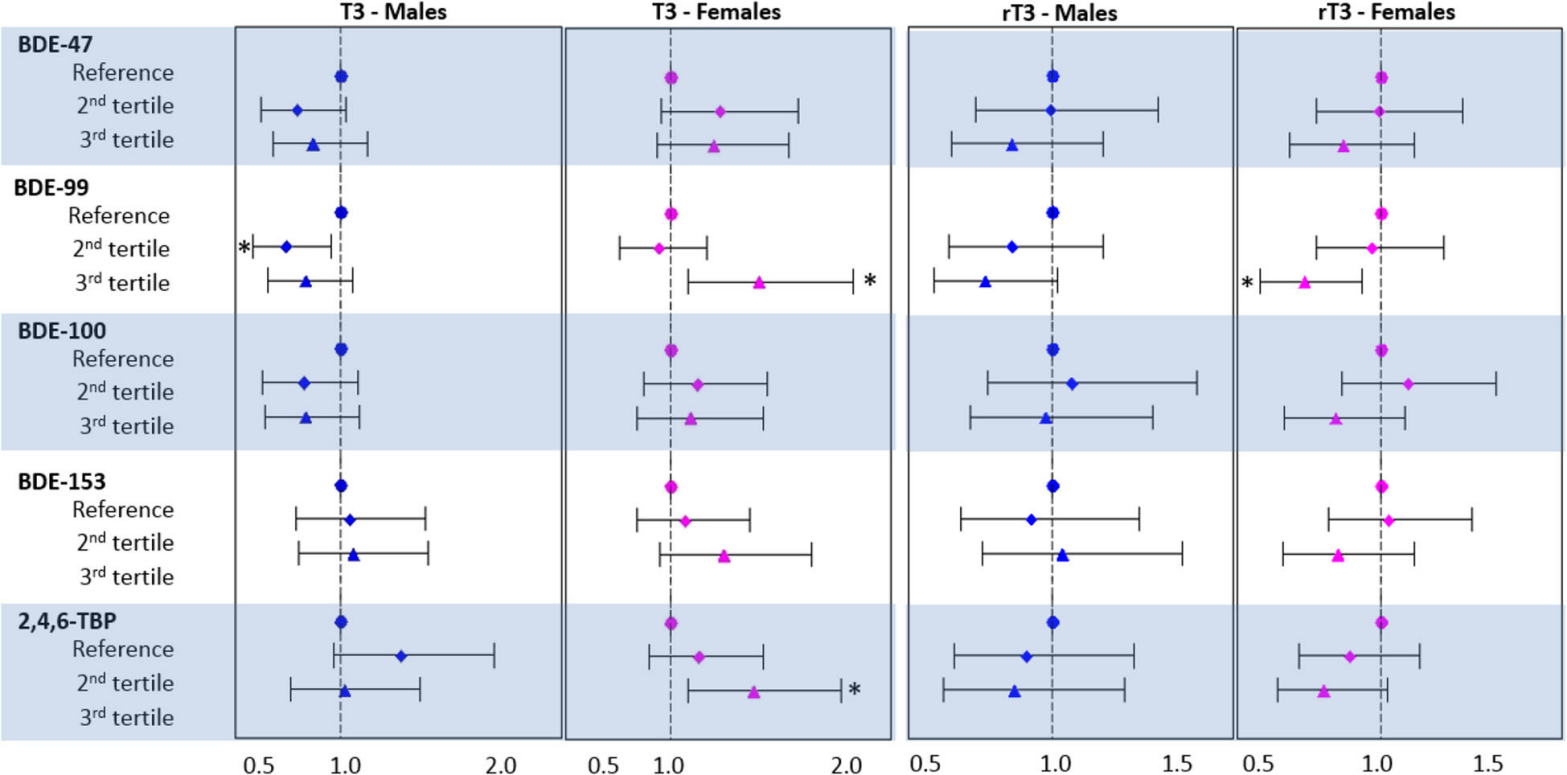
Multiplicative change in T3 and rT3 concentrations measured in placenta separated by tertile of each BFR. Analyses are adjusted for gestational age and maternal age. *Horizontal bars* reflect the 95% CI and * indicates *p* < 0.05

Adjusted regression analyses were also conducted to assess associations between BFRs and THs while adjusting for potential confounding by maternal age and gestational age at the time of delivery. As in correlation analyses, none of the BFRs were significantly associated with T3 in analyses including all participants. However, although results generally did not reach statistical significance, BDE-47, −99, and −100 were negatively associated with T3 concentrations among males, while BDE-47, −99, −100, −153 and 2,4,6-TBP were positively associated with T3 among females. For example, among males, those with the highest concentrations of BDE-99 in placenta had T3 levels 0.80 times those with the lowest concentration of BDE-99 (95% confidence interval (CI): 0.59, 1.07). Whereas females with the highest concentrations of BDE-99 in placenta had T3 levels 1.50 times those with the lowest concentration of BDE-99 (95% CI: 1.10, 2.04). We observed little evidence of associations between any of the BFR analytes and T4 concentrations; beta estimates were generally small, imprecisely estimated, and not statistically significant. These data are summarized in Fig. 2.

Among males, our results suggest that rT3 concentrations may be inversely related to BDE-47, −99, −209, and 2,4,6-TBP, with BDE-99 having the strongest association in the highest tertile (10^β^ = 0.72, CI: 0.51, 1.02). In females, rT3 was generally inversely associated with BDE-47, −99, −100, −153, and 2,4,6-TBP in the highest tertiles, with BDE-99 having the strongest association (10^β^ = 0.68, CI: 0.50, 0.92).

### DIO3 and SULT activities

DIO3 activity was measured in all placenta tissue microsome samples. The geometric mean value of DIO3 activity was 0.74 pmol rT3/mg protein/min, with a range of 0.01–3.86 rT3/mg protein/min (RSD = 6.9%). Interestingly, DIO3 activity was significantly higher (~1.8 times) in placental tissues from male infants compared to female infants (*p* < 0.01). DIO3 activity was negatively correlated with T4 concentrations (*r*_s_ = −0.21; *p* = 0.04); however, Spearman correlations were small and were not statistically significant between DIO3 activity and other BFRs. In regression analyses, DIO3 activity among males showed a suggestive positive association with BDE-99, −100, and −153; however, no relationships were statistically significant at the *p* < 0.05 level. In females, DIO3 activity tended to be negatively associated in the highest tertile for BDE-47, −99, −100, −153, and 2,4,6-TBP, with BDE-99 having the strongest association (10^β^ = 0.49, CI: 0.26, 0.91).

TH SULT activity was measured in all placenta tissue cytosol samples. Both 3,3’-T2 and T3 SULT activities were assessed in this study. The geometric mean value of 3,3’-T2 SULT activity was 3.32 pmol T2S/mg protein/min (range: 0.35–19.2 pmol T2S/mg protein/min; RSD = 5.7%), while the geometric mean value of T3 SULT activity was 9.50 fmol T3S/mg protein/min (range: 3.59–37.9 fmol T3S/mg protein/min; RSD = 6.8%). The 3,3’-T2 SULT enzyme activities were approximately one order of magnitude greater than the T3 SULT activities, which was expected based on their substrate preferences. 3,3’-T2 SULT activity was significantly higher (~1.5 times) in placental tissues from females compared to males (*p* < 0.01). There was no observed sex difference for T3 SULT activity in placental tissues.

We observed a negative correlation between BDE-209 and 3,3’-T2 SULT activity (*r*_s_ = −0.19; *p* = 0.06) in analyses using all samples, although the magnitude of the correlation was small and not statistically significant. 3,3’-T2 SULT activity was not associated with other BFRs. Among male infants, 3,3’-T2 SULT activity was positively associated with rT3 (*r*_s_ = 0.29; *p* = 0.04). In combined analyses (males and females) T3 SULT activity was not associated with any BFR analytes. However, among males, T3 SULT activity was positively associated with BDE-47 (*r*_s_ = 0.29; *p* = 0.04) and showed positive, but non-statistically significant associations with BDE-100, −153, and ΣBDEs. There were no statistically significant associations found in the female group; however, there was a negative trend between BDE-99 and T3 SULT. Regression analyses produced similar results. For example, T3 SULT activity among males was positively associated with BDE-47, −100, and −153, with BDE-153 having the strongest association (10^β^ = 1.48, CI: 1.05, 2.09 comparing the 3^rd^ to 1^st^ tertile). As in correlations analyses, T3S activity in females showed a negative association with BDE-99 (10^β^ = 0.67, CI: 0.49, 0.91) in adjusted regression models.

## Discussion

The goals of this study were to measure TH tissue concentrations and endogenous TH DIO3 and SULT activity in placenta tissue subsamples and to examine their associations with BFRs. THs are essential to both fetal and placental development. For example, THs are important in trophoblast proliferation and migration during placenta development, and restrictions on TH delivery to the placenta can lead to several disorders of the placenta [21]. In addition, maternal thyroid disorders have been associated with pre-eclampsia, placental abruption, and intra-uterine growth restriction (IUGR) [32–35]. Therefore, understanding environmental stressors that may affect thyroid regulation in the placenta is of critical importance.

To our knowledge, this is the first study to measure T4, T3, and rT3 in human placental tissues using liquid chromatography tandem mass spectrometry (LC/MS-MS), and the first study to measure 3,3’-T2/T3 SULT activity in human placental tissues. The use of LC-MS/MS offers greater specificity and sensitivity over RIA-based methods [30]. To our knowledge, THs have only been measured in placenta in one previous study in a cohort of 16 women with normal pregnancies using an RIA-based method. This study reported values for T4, T3, and rT3 (18.8, 0.03, and 1.70 ng/g tissue, respectively) [24], similar to levels reported here. Serum T4/T3 ratios are widely used in the clinical setting to evaluate TH status in individuals; however, the measurement of THs in specific tissues is not widely used, and there is not much known about the TH status of specific tissues such as the placenta, nor how it changes during pregnancy.

In general, our results suggest that the concentrations of BFRs in the placenta may be associated with lower levels of rT3. Associations were also observed for T3, but differed by sex (negative associations for males and positive associations for females). Although PBDEs were not measured in placenta in past studies, previous pregnancy studies have reported positive, negative, and no associations between serum PBDE and TH concentrations. For example, in a recent study by Abdelouahab et al., PBDEs and THs were measured in maternal blood and umbilical cord blood at two different time points during gestation [36]. That study observed negative associations between PBDEs and Total T4 (TT4) and Total T3 (TT3), as well as positive associations between PBDEs and free T4 and free T3 in maternal serum collected at less than 20 weeks gestation. However, these associations reversed for free T4 and free T3 in maternal blood collected at the time of delivery [36]. A 2010 study by Stapleton et al. observed positive associations between PBDEs and TT3, TT4, and free T4 in maternal blood samples collected during the third trimester [27]. Finally, several studies have reported no associations between PBDEs and THs measured in maternal serum samples collected during the second trimester, collected at delivery, or in umbilical cord blood [37–39]. Past studies and our own work may not be directly comparable due to differences in their experimental designs and methods. Additionally, inter-individual variability in TH set-points around which TH homeostasis operates may contribute variability to these studies. These differences are magnified during pregnancy, and this contributes to the difficulty in determining a normal TH profile for a pregnant mother (serum concentrations), and perhaps within the placenta tissue itself [40].

We observed statistically significant negative associations between rT3 and BDE-99 tissue concentrations in all samples. rT3 is the genomically inactive form of TH that is formed from the inner ring deiodination (IRD) of T4 by type 1 DIO (DIO1) or DIO3. DIO3 is one of three DIO enzyme isoforms that only performs IRD. The placenta exhibits high DIO3 expression in early pregnancy, and total DIO3 activity increases significantly throughout gestation until birth [41]. It is thought that DIO3 plays an important role in buffering the maternal supply of T4 within the placental tissue compartment before being transported to fetal circulation, as well as provides a source of iodide for the fetus via deiodination [21, 22]. As a result, DIO enzyme inhibition by environmental contaminants such as PBDEs and their metabolites may lead to reductions in T4 deiodination, causing higher levels of T4 and lower levels of rT3 in placental tissues. Such inhibition, particularly by BDE-99, has been shown using in vitro models with human liver cytosol and human glial cells [12, 42]. This mechanism may contribute to the observations in this study, as higher concentrations of BDE-99 were associated with lower DIO3 activity and lower concentrations of rT3 among females. However, among males, we observed inverse trends between BDE-99 and rT3 in the absence of an impact on DIO3, suggesting an alternate mechanism of action between BDE-99 and rT3.

Our current work is the first to evaluate TH SULT activity in human placental tissues. We chose to evaluate SULT activity because in vitro work from our laboratory using pooled human liver cytosol indicated that TH SULT enzymes are more sensitive to inhibition by BFRs compared to DIO activity [11]. However, 3,3’-T2 SULT activity did not show any statistically significant associations with BFRs. The variability in 3,3’-T2 SULT activity amongst males is much higher than among females and may provide some insight into the sex differences in placental SULT expression and activity.

T3 SULT activity was also measured in the samples. The T3 SULT enzyme activity rates in this study were a full order of magnitude lower than that of 3,3’-T2. These results are consistent with other studies that have compared the sulfation rates of the different iodothyronines in various tissues types, with 3,3’-T2 being the preferred substrate for sulfation and having an activity rate 1–2 orders of magnitude greater than T3 [43–45]. As such, 3,3’-T2 is used as a surrogate model for T3 sulfation kinetics in in vitro experiments [46]. The positive association between 3,3’-T2 and T3 SULT activity observed here supports this claim. However, T3 sulfation plays a greater biological role in regulating the available pool of T3 for subsequent binding to thyroid receptors [47]. Sulfation of T3 is a reversible reaction that inactivates the biological activity of T3 and is thought to create a reservoir of T3 that can be reactivated following hydrolysis by arylsulfatases in a tissue-specific manner [21]. This metabolic pathway is a potentially important step for buffering the concentrations of T3 in the placenta before delivery to the fetal compartment. In this study, T3 SULT enzyme activities showed a significantly positive association with BDE-47 in males (*r*_s_ = 0.29, *p* = 0.04), and a suggestive negative association with BDE-99 in females (*r*_s_ = −0.25, *p* = 0.09). Additionally, we observed a positive association for the highest tertile of BDE-153 exposure in males (10^β^ = 1.48, *p* = 0.03), and a negative association in the highest tertile of BDE-99 exposure in females (10^β^ = 0.67, *p* = 0.01) in our adjusted models. This may suggest that BDE-99 is inhibiting T3 sulfation in placental tissues and leading to an increase in T3; however, it is unclear why this was only observed in placental tissues from females.

One interesting finding from this study is that BFR levels in placenta, and their associated impacts, may be dependent upon infant sex. Despite seeing no difference in maternal serum levels, BFR levels in placenta were higher in placenta samples associated with males. The underlying mechanisms of these sex-specific differences are not known at this time; however, the sex of the infant seems to play a role in the bioaccumulation potential, metabolism, and/ or kinetic parameters of the placenta during pregnancy. It is possible that the sex-specific differences in placental morphology and function may play a role in this organ’s ability to accumulate and metabolize BFRs [48]. While other studies have examined PBDE accumulation in placental tissues, no studies to date have identified differences based on infant sex. This may be due to the smaller sample size of these studies, which were generally less than 50 samples, or due to the fact that they were conducted in regions where use and exposure to PBDEs was lower than in the U.S. (e.g. Europe). Initially it was suspected that differences in lipid content between male and female placentae were driving differences in bioaccumulation of BFR compounds; however, there were no observed differences in tissue total lipid content between sexes. It is still possible that fine-scale differences in the lipid profiles between sexes may be driving these observed BFR bioaccumulation rates, and future research should utilize a lipidomic assessment of placenta tissue in order to fully characterize the lipid molecule profile of placental tissues. This is important because PBDEs have been shown to differentially bind to various classes of lipoproteins, and there may exist sex differences in the expression of placenta lipoproteins [49, 50]. One additional explanation of differences in BFR bioaccumulation rates may be that the placental tissue expression of TH transporting membrane proteins, including monocarboxylate transporters (MCTs), System-L amino acid transporters (LATs), and organic-anionic transport protein (OATPs), is sexually dimorphic. Higher expression levels of these transporter proteins in male placentae may lead to higher rates of transport of TH-like compounds such as PBDEs and consequently, higher tissue concentrations [51, 52].

This is also the first study to compare placental tissue concentrations of THs with BFR concentrations in a human cohort, as well as one of the first to measure placental tissue DIO3 and SULT activity. THs are transported in the blood bound to transport proteins such as TTR and TBG before uptake into cells in peripheral tissues via TH transport proteins such as MCTs, LATs, and OATPs [53]. However, little is known about the tissue concentrations of THs after uptake and subsequent metabolism by DIOs or SULTs. The tissue concentrations of THs are important in facilitating TH-mediated genomic activity, and in the case of the placenta, important in regulating the maternal supply of THs to fetal circulation to support growth. The results of this study indicate that placental TH concentrations may be sensitive to BFR tissue concentrations in a sex-specific manner, and may have possible downstream effects on TH-mediated developmental processes throughout gestation.

A limitation of this study is the nature of the placenta tissue subsamples. First, the tissues may not have been consistently harvested from the same sections of the organ following delivery. This means that some tissue subsamples may have been taken from the peripheral sections of the placenta, while others may have been taken from more central locations. Due to the heterogeneous nature of the placenta, as well as spatial differences in cell type, vasculature, and lipid content of the organ, the samples analyzed in this study may not be equally representative. Although we measured BFRs, THs and enzyme activity in 95 samples, our analyses were also limited by our relatively small sample size which may have reduced our statistical ability to detect meaningful associations, particularly after stratification. In addition, we evaluated the impact of several potential confounding variables in assessing associations; nonetheless, it is possible that our results may be explained by residual confounding by other unmeasured variables; however, our cohort was relatively homogeneous, with the majority of the women reporting African American race and low educational attainment. While this may reduce the potential for confounding bias it could also limit the generalizability of our findings to other study populations. In addition, a comparison of maternal TH levels and placenta levels would be of great interest. Unfortunately we did not have paired maternal samples with TH measurements for women in our cohort and we were unable to evaluate relationships; however, future studies should consider including a comparison of TH measurements between the maternal and placental compartments. Additionally, future studies should evaluate TH concentrations, as well as other TH-related endpoints within the placenta tissue in order to broaden our understanding of BFR effects on TH homeostasis during pregnancy.

## Conclusions

Cumulatively, our research suggests BFRs accumulate in the placenta, and may be associated with TH changes in a sex-specific manner. More research is needed to elucidate the effects of BFRs within placenta during pregnancy, as well as the mechanisms of action and biological consequences of exposure; however, our results provide a possible mechanism to support sex-differences reported for the impacts of environmental exposure on children’s growth and development.

## Additional file

Additional file 1: Supplemental material. (DOCX 283 kb)

## Abbreviations

2,4,6-TBP: 2,4,6-tribromophenol
3,3’-T2: 3,3’-diiodothyronine
3,3’-T2S: 3,3’-diiodothyronine sulfate
DI: Deiodinase
DIO3: Type 3 deiodinase
LC-MS/MS: Liquid chromatography tandem mass spectrometry
PBDE: Polybrominated diphenyl ether
rT3: Reverse triiodothyronine
SULT: Sulfotransferase
T3: Triiodothyronine
T3S: 3,3’,5-triiodothyronine sulfate
T4: Thyroxine
TBG: Thyroid binding globulin
THs: Thyroid hormones
TTR: Transthyretin
