# Supplementary material for: Brominated flame retardants in placental tissues: associations with infant sex and thyroid hormone endpoints
Source: Environ Health. 2016 Nov 25;15:113. doi: 10.1186/s12940-016-0199-8 (PMC5123327; doi:10.1186/s12940-016-0199-8)
Supplement: Additional file 1: — Supplemental material. (DOCX 283 kb) [file 12940_2016_199_MOESM1_ESM.docx]

**Supplemental Material**

**Associations between Brominated Flame Retardants, Thyroid Hormone Levels, and Thyroid Hormone Regulating Enzyme Activity in Human Placental Tissues**

Christopher Leonetti^1^, Craig M. Butt^1^, Kate Hofman^1^, Marie Lynn Miranda^2^, and Heather M. Stapleton^1^

1. Nicholas School of the Environment, Duke University, Durham, North Carolina, USA
2. Department of Statistics, Rice University, Houston, TX, USA

**Corresponding Author:**

Heather M. Stapleton, Ph.D.

Duke University

Nicholas School of the Environment

9 Circuit Drive, Box 90328

Durham, NC 27708

Phone: 919-613-8717

Email: heather.stapleton@duke.edu

**Contents**

Reagents and Materials…………………………………………………………………………..S3

Placenta BFR and TH Analysis………………………………………………………………….S4

Preparation of Placental Microsomes……………………………………………………………S5

Deiodinase Activity Assays………………………………………………………………...……S6

Deiodinase Activity Assays………………………………………………………………….…..S7

Sulfotransferase Assays ………………………………………………………………...……….S8

Instrumental Analysis………………………………………………………………..…..………S9

Figure S1 – Forest plot for T_4_……………………………………………………………..……S10

Figure S2 – Forest plot for DIO3…………………………………………………….…………S11

Figure S3 – Forest plot for T_3_ SULT…………………………………………...………………S12

Figure S4 – Forest plot for 3,3’-T2 SULT………………………………………………...……S13

Table S1 – Spearman correlation coefficients …………………………………………………S14

*Reagents and Materials*

Isotopically labeled thyroid hormone standards (^13^C_12_- T_4_, ^13^C_6_- T_4_, ^13^C_6_-T3, and ^13^C_6_-rT3) were used as internal and recovery standards for the TH extractions were purchased from Isotec (Miamisburg, OH). Dithiothreitol (DTT, > 99%), 3′-phosphoadenosine 5′-phosphosulfate (PAPS), T_4_ (> 98%), T_3_ (> 95%), and rT3 (> 95%), were purchased from Sigma-Aldrich (St. Louis, MO). 3,3’,5-triiodothyronine sulfate (> 95%) was purchased from Toronto Research Chemicals (Toronto, Ontario). ^13^C_6_-3,3′-T2 was purchased from Isotec (Miamisburg, OH). 3,3′-T2 sulfate (3,3′-T2S, 98%) was custom synthesized by the Duke University Small Molecule Synthesis Facility (Durham, NC). 3,3’,5- T_3_ sulfate (T_3_S, > 98%) was purchased from Toronto Research Chemicals (Toronto, ON). All solvents and other reagents were purchased from VWR (Radnor, PA).

*Placenta BFR and Thyroid Hormone Analysis*

Placenta tissue samples were analyzed for BFR and lipids as described in our previously published work (Leonetti et al. 2016). Placenta tissue samples (approximately 200 milligrams) were analyzed for thyroid hormones were using a modified extraction protocol from our laboratory. Briefly, tissues were homogenized using 0.5 mm glass beads and a BBX24 Bullet-Blender, prior to a 16 hour protein/tissue digestion in a 37°C water bath. The pronase digestion solution contained protease (*Streptomyces griseus*), L-glutathione, n-phenylthiourea, and tris hydroxymethyl aminomethane. Samples were spiked with a mixture of 5 ng of ^13^C_12_- T_4_, ^13^C_6_-T3, and ^13^C_6_-rT3 as internal standards. Following digestion, 40 μL of an antioxidant solution containing 37.5 mg/mL of citric acid, ascorbic acid, and dithiothreitol were added to each sample, followed by 500 μL of cold acetone. Samples then undergo a series of solvent extractions and centrifugation in order to separate THs and remove lipids and other competing biomolecules. Samples are extracted sequentially using acetone, cyclopentane, and ethyl acetate. After extraction, the samples are concentrated by reducing the solvent volume to 50 uL under a stream of N_2_ gas and resupended in 3 mL of 0.01 M HCl with 10% MeOH. Next, samples undergo clean-up/extraction via solid-phase extraction (SPE) using SampliQ OPT cartridges to further remove competing biomolecules and isolate the analytes of interest (T_4_, T_3_, and rT3). Cartridges are conditioned with 3 mL of MeOH, followed by 3 mL of H_2_O, and then samples are loaded. Cartridges are then washed with 20% MeOH in H_2_O and analytes were eluted in 4 mL of 0.1% acetic acid in MeOH and blown down under a stream of N_2_ gas to a final volume of 50 uL. Samples were resuspended in 400 μL of 1:1 H_2_O/MeOH, transferred to glass screw-top ASV vials, and spiked with 5 ng of ^13^C_6_-T_4_ as a recovery standard. Finally, THs are identified and quantified using authenticated standards and liquid chromatography tandem mass spectrometry (LC-MS/MS) with electrospray ionization (ESI).

For the TH data, MDLs are calculated as three times the standard deviation of the blank values for each analyte (T_3_, T_4_, and rT3). Individual values are then normalized to the wet tissue mass used for the extraction procedure to yield a final value of ng/g wet weight (ww).

*Preparation of Placental Microsomes*

For each placenta sample, approximately 4 g of tissue was sub-sampled, avoiding the fatty tissue, minced and homogenized using a Bullet Blender (Next Advance, Averill Park, NY) with stainless stain beads (1:2:1 ratio of 0.2 mm:0.5 mm: 1.4 mm diameter beads). Microsomal suspensions were prepared from the homogenized placental tissues using methods adapted from McKinney et al (McKinney et al. 2004). Briefly, the homogenization buffer consisted of 0.25 M sucrose, 0.1 M KPO4, 1 mM EDTA, 10 mM DTT (pH 7.4). The microsome resuspension buffer was prepared identically to the homogenization buffer with the addition of 20% glycerol. All solution were prepared fresh daily. Care was taken to ensure that all preparations were done on ice to preserve enzyme integrity. Protein concentrations were determined using the Pierce™ BCA Protein Assay Kit (Thermo Scientific, Rockford, IL) with bovine serum albumin as the protein standard. Prepared microsomes were kept frozen at -80C until use in the experimental assays.

*Deiodinase Activity Assays*

Deiodinase activity assays were performed using *in vitro* techniques and LC-MS/MS analysis as described by Butt et al., 2011. Briefly, human placenta microsomes were diluted to approximately 1 mg protein/ml in 0.1 M potassium phosphate buffer (pH 7.4) with 10 mM DTT and 100 µM NADPH (total volume = 1 ml). In addition, buffer controls were prepared to correct for substrate impurities and abiotic degradation. Assays were predominately performed in triplicate (n=75) with the remaining run in duplicate (n=11) or single measurement (n=16). Reactions were initiated by the microsome addition and incubated at 37^o^C for 1 hr in a shaking water bath. Assays were stopped with 1 ml of ice-cold methanol, spiked with a suite of mass-labelled internal standards (25 ng each of ^13^C_12_-T_4_, ^13^C_6_-T_3_, ^13^C_6_-rT3 and ^13^C_6_-3,3’-T2) and extracted using liquid-liquid extraction. The organic solvent was evaporated under a gentle stream of nitrogen gas and the extracts were cleaned and concentrated using solid-phase extraction (SPE) techniques. The SPE eluent was reduced to approximately 1 ml and analyzed for THs by LC-MS/MS. Analyte responses were normalized to the corresponding internal standard responses.

Prior to performing the placental DI activity study, the experimental conditions were optimized by varying the microsomal protein concentration and incubation time. The results showed that the optimized in vitro parameters (i.e. 1 hr incubation time, 1 mg/ml protein concentration) were within the linear range of the variables (shown below in **Figure S1** and **Figure S2)**.

**Figure S1.** DI assay optimization – incubation time.

**Figure S2.** DI assay optimization – microsomal protein concentration.

*Sulfotransferase Assays*

Placenta tissue cytosol was created during microsome preparation. Approximately 6 mL of cytosol were produced per tissue sample and stored at -80 °C to ensure enzyme integrity until analysis. TH SULT activity was measured using a previously published SULT assay (Butt and Stapleton 2013). Briefly, placenta cytosol was combined with 0.1 M potassium phosphate buffer (pH=7.2) with 50 μM PAPS as the cofactor and 1 μM 3,3’-T2 or T_3_ as the substrate (total volume of 200 μL). Assay reactions were started with the addition of the cytosol. Vials were incubated at 37 °C for 30 minutes in a shaking water bath, and reactions were stopped by the addition of 0.1 M HCl (800 μL). Samples were then spiked with ^13^C_6_-3,3′-T2 (5.0 ng) or ^13^C_6_-T_3_ (5 ng) as the internal standard. Sample extracts were cleaned using SampliQ OPT SPE cartridges (Agilent Technologies). The SPE columns were first conditioned with 3 mL of methanol and 3 mL of water, samples were loaded, and the column was rinsed with 3 mL of water. The THs and sulfate conjugate were eluted with 4 mL of methanol, and the extract was reduced to approximately 50 μL under a gentle stream of nitrogen gas. Samples were reconstituted in 1:1 MeOH/H_2_O and transferred to Mini-UniPrep Syringeless Filters (GE Life Sciences) before analysis by LC-MS/MS. The protein content of cytosolic fractions was determined using the Bradford assay and SULT activity measurements were calculated as picomoles of 3,3’-T2 or femtomoles of T_3_ formed per minute per milligram of protein.

*Instrumental Analysis*

Instrumental analysis was performed by liquid chromatography with electrospray ionization (ESI) tandem mass spectrometry (LC−MS/MS) using conditions modified from our previously published methods. Monitored analytes included 3,3′-T2, 3,3′-T2S, and T_3_S. The sulfated THs was analyzed in electrospray ionization negative mode. MS/MS parameters for 3,3′-T2, 3,3′-T2S, and T_3_S were optimized using authentic standards. All analyte responses were normalized to the response of ^13^C_6_-3,3′-T2.


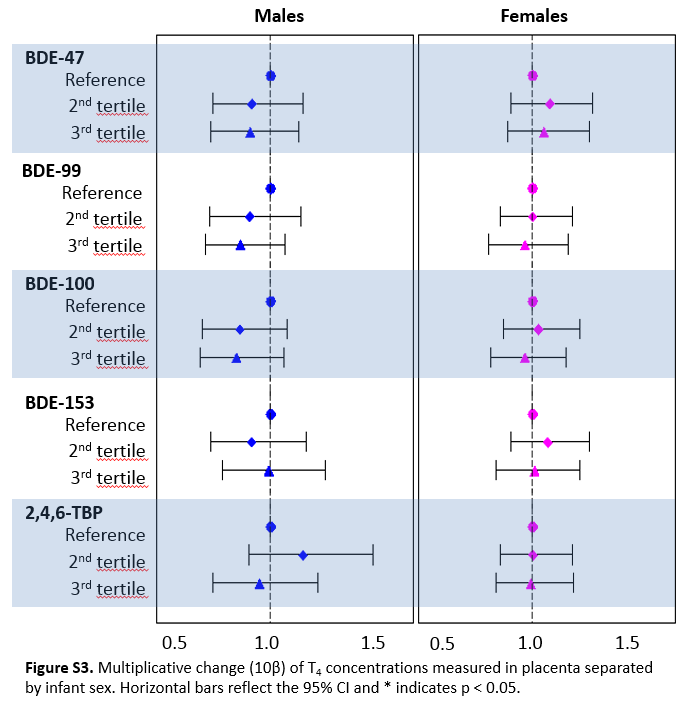


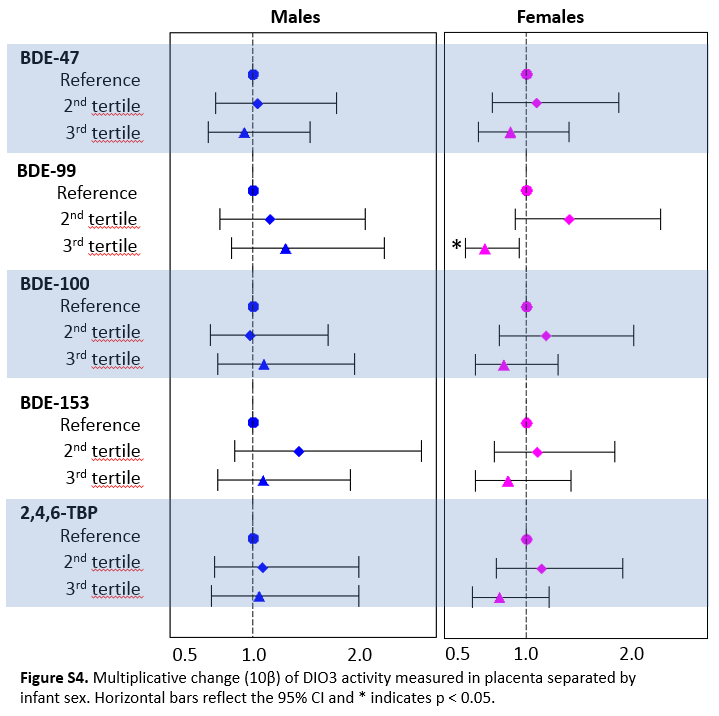


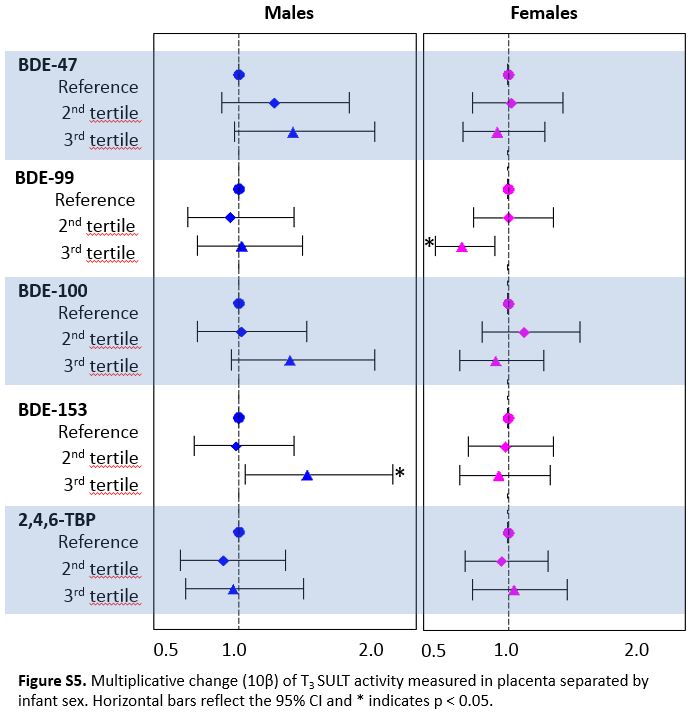


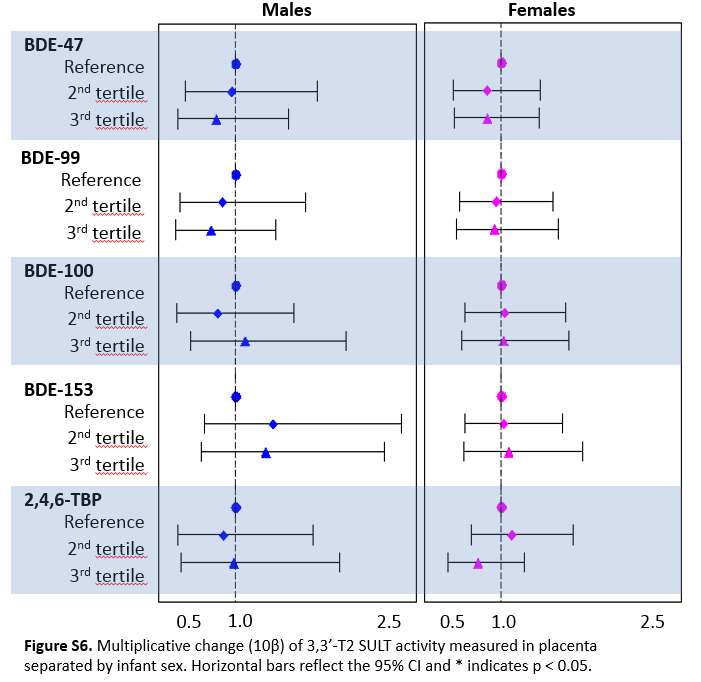


|  | **BDE-47** | **BDE-99** | **BDE-100** | **BDE-153** | **BDE-154** | **BDE-209** | **2,4,6-TBP** | **ΣBDEs** | **ΣBFRs** | **T3** | **rT3** | **T4** | **DIO3** | **T2 SULT** | **T3 SULT** |
| --- | --- | --- | --- | --- | --- | --- | --- | --- | --- | --- | --- | --- | --- | --- | --- |
| **BDE-47** | 1.00 |  |  |  |  |  |  |  |  |  |  |  |  |  |  |
| **BDE-99** | 0.46^#^ | 1.00 |  |  |  |  |  |  |  |  |  |  |  |  |  |
| **BDE-100** | 0.87^#^ | 0.50^#^ | 1.00 |  |  |  |  |  |  |  |  |  |  |  |  |
| **BDE-153** | 0.58^#^ | 0.44^#^ | 0.72^#^ | 1.00 |  |  |  |  |  |  |  |  |  |  |  |
| **BDE-154** | 0.61^#^ | 0.50^#^ | 0.72^#^ | 0.75^#^ | 1.00 |  |  |  |  |  |  |  |  |  |  |
| **BDE-209** | 0.46^#^ | 0.58^#^ | 0.47^#^ | 0.49^#^ | 0.52^#^ | 1.00 |  |  |  |  |  |  |  |  |  |
| **2,4,6-TBP** | 0.48^#^ | 0.64^#^ | 0.47^#^ | 0.39^#^ | 0.46^#^ | 0.54^#^ | 1.00 |  |  |  |  |  |  |  |  |
| **ΣBDEs** | 0.83^#^ | 0.67^#^ | 0.88^#^ | 0.78^#^ | 0.78^#^ | 0.71^#^ | 0.56^#^ | 1.00 |  |  |  |  |  |  |  |
| **ΣBFRs** | 0.72^#^ | 0.71^#^ | 0.77^#^ | 0.68^#^ | 0.71^#^ | 0.69^#^ | 0.83^#^ | 0.89^#^ | 1.00 |  |  |  |  |  |  |
| **T3** | 0.07 | 0.06 | 0.00 | 0.10 | -0.04 | 0.01 | 0.13 | 0.04 | 0.07 | 1.00 |  |  |  |  |  |
| **rT3** | -0.05 | -0.30* | -0.02 | 0.03 | -0.02 | -0.15 | -0.05 | -0.14 | -0.08 | -0.01 | 1.00 |  |  |  |  |
| **T4** | -0.05 | -0.16 | -0.14 | -0.02 | -0.09 | -0.08 | -0.05 | -0.11 | -0.08 | 0.49^#^ | 0.26* | 1.00 |  |  |  |
| **DIO3** | 0.08 | 0.10 | 0.19 | 0.09 | 0.11 | 0.15 | 0.10 | 0.15 | 0.13 | -0.10 | 0.18 | -0.21* | 1.00 |  |  |
| **T2 SULT** | 0.01 | -0.14 | -0.02 | 0.01 | 0.03 | -0.19 | -0.16 | -0.03 | -0.11 | -0.14 | 0.08 | -0.09 | -0.09 | 1.00 |  |
| **T3 SULT** | 0.16 | -0.11 | 0.14 | 0.18 | 0.13 | 0.00 | -0.05 | 0.09 | 0.02 | -0.11 | 0.03 | 0.03 | 0.13 | 0.24* | 1.00 |
| **Table S1**. Spearman correlation coefficients for BFRs, THs, and DIO3 and SULT activity in placenta tissue samples (n=95; combined data, both male and female infants). | | | | | | | | | | | | | | | |
